# Construction and validation of a clinical risk model based on machine learning for screening characteristic factors of lymphovascular space invasion in endometrial cancer

**DOI:** 10.1038/s41598-024-63436-7

**Published:** 2024-06-01

**Authors:** Fang Wang, Rui Pang, Shaohong Shi, Yang Zhang

**Affiliations:** Department of Gynaecology, Xuzhou Medical University Affiliated Hospital of Lianyungang, No. 6, Zhenhua East Road, Lianyungang, 222061 Jiangsu Province China

**Keywords:** Endometrial cancer, Lymphovascular space invasion, Machine learning, Logistic regression, LASSO regression, Cancer, Gynaecological cancer

## Abstract

This study aimed to identify factors that affect lymphovascular space invasion (LVSI) in endometrial cancer (EC) using machine learning technology, and to build a clinical risk assessment model based on these factors. Samples were collected from May 2017 to March 2022, including 312 EC patients who received treatment at Xuzhou Medical University Affiliated Hospital of Lianyungang. Of these, 219 cases were collected for the training group and 93 for the validation group. Clinical data and laboratory indicators were analyzed. Logistic regression and least absolute shrinkage and selection operator (LASSO) regression were used to analyze risk factors and construct risk models. The LVSI and non-LVSI groups showed statistical significance in clinical data and laboratory indicators (*P* < 0.05). Multivariable logistic regression analysis identified independent risk factors for LVSI in EC, which were myometrial infiltration depth, cervical stromal invasion, lymphocyte count (LYM), monocyte count (MONO), albumin (ALB), and fibrinogen (FIB) (*P* < 0.05). LASSO regression identified 19 key feature factors for model construction. In the training and validation groups, the risk scores for the logistic and LASSO models were significantly higher in the LVSI group compared with that in the non-LVSI group (*P* < 0.001). The model was built based on machine learning and can effectively predict LVSI in EC and enhance preoperative decision-making. The reliability of the model was demonstrated by the significant difference in risk scores between LVSI and non-LVSI patients in both the training and validation groups.

## Introduction

Endometrial cancer (EC) is a common malignancy that has increased in incidence in women worldwide and has a serious impact on patient quality of life^1^. EC is primarily classified into two types: I and II. Of these, type I EC is commonly found in obese women, is associated with hyperlipidemia and hyperestrogenism, and is estrogen-sensitive, with a slow course of disease and a good prognosis^2^. Type II EC progresses rapidly and is associated with a poor prognosis, but is not significantly related to estrogen. The 5-year overall survival (OS) of early-stage EC patients usually exceeds 80%, but the prognosis decreases significantly when lymph nodes are involved^3^. Standard treatment for EC includes total hysterectomy, bilateral adnexectomy, and lymph node evaluation, with the latter focusing on pelvic and para-aortic lymph nodes^4^. The risk assessment of lymph node metastasis is critical for surgical planning; however, the indication for lymphadenectomy in patients with early-stage EC is controversial^5^. Lymphadenectomy in low-risk patients may not significantly affect progression-free survival, but increases the risk of surgical complications^6^. Conversely, for intermediate- and high-risk patients, lymphadenectomy may help prolong progression-free survival^7^. Therefore, preoperative risk stratification is important for deciding whether to proceed with lymphadenectomy.

The European Society for Medical Oncology (ESMO)-modified classification, the Mayo model, and the GOG-99 model are commonly used to assess the low-risk classification of EC patients^8^. Of these, the ESMO-modified classification was shown to have the highest accuracy in predicting lymph node metastasis^9^, indicating that this model is a reliable tool that can help physicians more accurately assess patient risk of lymph node metastasis. The ESMO classification regards lymphovascular space invasion (LVSI) as a key factor for early-stage EC in risk stratification^10^, which closely links the presence of LVSI to the risk of lymph node metastasis in patients. Although the diagnosis of LVSI depends on postoperative pathological analysis, there are currently no effective biomarkers to determine LVSI status before or during surgery^11^. The intraoperative frozen section technique was also limited by time and sample size in the detection of LVSI, making it difficult to make an accurate identification^12^. Therefore, predictive models, as a statistical tool, have been widely used to simplify the clinical prediction process by graphing complex regression equations^13^. Combined predictive models have been used to estimate LVSI risk, and have demonstrated some accuracy^14,15^; however, the existing prediction models for LVSI in EC are limited and mainly constructed based on pathological indexes.

Through the application of machine learning technology, this study aimed to screen out the key characteristic factors affecting LVSI in EC and build a clinical risk assessment model based on these factors. The construction and validation of this model may be of significance for improving the accuracy of LVSI prediction and clinical decision-making, while providing more accurate prognostic information for high-risk patients, with potentially important clinical implications.

## Methods and materials

### Ethical statement

This retrospective study complied with the Declaration of Helsinki and was approved by the Ethics Committee of the Affiliated Hospital of Xuzhou Medical University, Lianyungang (2022-03). Because of the retrospective nature of this study, informed consent was waived with the approval of the Ethics Committee of the Affiliated Hospital of Xuzhou Medical University, Lianyungang.

### Eligibility and exclusion criteria

Inclusion criteria: Patients who received treatment at the Xuzhou Medical University Affiliated Hospital of Lianyungang for the first time and were confirmed as EC by postoperative pathology. The surgical treatment included either total extra-fascial hysterectomy or radical hysterectomy plus bilateral adnexectomy, which may be supplemented by peritoneal irrigation, pelvic lymphadenectomy, and/or para-aortic lymphadenectomy as appropriate. The patient did not receive radiotherapy, chemotherapy, targeted therapy, or hormone therapy before surgery. The pathological findings of the patient’s preoperative endometrial biopsy (including pathological types and histological grades), myometrial infiltration (determined by pelvic MRI), and tumor diameter (detected by hysteroscopy or gynecological color Doppler ultrasound) were consistent with the postoperative pathological results.

Exclusion criteria: Patients with malignancies in other systems, a history of coagulation dysfunction, autoimmune diseases, severe liver and kidney dysfunction, or those with incomplete clinical medical records were excluded.

### Sample source

This study enrolled EC patients who received treatment at Xuzhou Medical University Affiliated Hospital of Lianyungang from May 2017 to March 2022.

### Sample screening

A total of 408 eligible samples were screened according to the inclusion criteria, and 312 samples were enrolled after removing samples based on the exclusion criteria.

### Sample grouping

The enrolled patients were divided into a training group (n = 219) and a validation group (n = 93) at a 7:3 ratio. They were subsequently divided into an LVSI group and a non-LVSI group based on the occurrence of LVSI. In the training group, there were 163 non-LVSI patients and 56 LVSI patients, whereas the validation group consisted of 72 non-LVSI patients and 21 LVSI patients.

### Outcome measures

1. The differences in clinical data and laboratory indicators between the LVSI and non-LVSI groups in the training group were compared. 2. Logistic regression was used to analyze the risk factors of LVSI in EC. 3. Least absolute shrinkage and selection operator (LASSO) regression was used to screen the characteristic factors of LVSI in EC. 4. Risk models were established using logistic regression and LASSO regression, and the difference in risk scores and predictive efficacy between LVSI and non-LVSI patients in the training group of the two models was compared. 5. The clinical data and laboratory indicators were compared between the training and validation sets, and the difference in risk scores and predictive performance between the two models in the validation set were calculated.

### Statistical analyses

The data were processed using SPSS 20.0 software. Data distribution was analyzed by the Kolmogorov–Smirnov (K–S) test. Measured data were described by means ± SD; the inter- and intra-group comparisons of normally distributed data were done by independent samples t-tests and paired t-tests (expressed by t), respectively; Whereas data conforming to a non-normal distribution were analyzed by nonparametric tests, specifically the Wilcoxon rank-sum test (expressed by Z), and are expressed by the median and interquartile distance (IQR). The comparison of counting data was done by χ^2^ tests. Multivariable logistic regression analysis was done to analyze the independent risk factors for LVSI in EC patients. The characteristic factors of LVSI in EC were screened by LASSO regression. Receiver operating characteristic (ROC) curves were plotted to analyze the value of the two models in predicting LVSI in EC, and the Delong test was used for comparison. *P* < 0.05 was considered statistically significant.

## Results

### Comparison of patient clinical data

Significant statistical differences were observed in myometrial infiltration depth, tumor diameter, pathological type, histological grade, and cervical interstitial involvement between the LVSI and non-LVSI groups when analyzing patient data (*P* < 0.05, Table 1).

**Table 1:** Analysis of the difference in clinical data between LVSI and non-LVSI groups in the training group.

Factors	Non-LVSI group (n = 163)	LVSI group (n = 56)	t	*P*
Age (years)	56.74 ± 9.14	59.38 ± 8.17	− 1.909	0.058
BMI (kg/m^2^)	25.85 ± 2.75	25.56 ± 1.69	0.726	0.468
Menarche (years of age)	14.06 ± 1.55	13.71 ± 1.22	1.518	0.130
Menopause				
Yes	55	15	0.928	0.336
No	108	41		
Gravidity				
≥ 2	104	41	1.650	0.199
< 2	59	15		
Parity				
Multipara	127	42	0.201	0.654
Primipara	36	14		
History of hypertension				
With	46	20	1.112	0.292
Without	117	36		
History of diabetes				
With	44	21	2.204	0.138
Without	119	35		
Myometrial infiltration depth (cm)				
< 1/2	134	22	37.474	< 0.001
≥ 1/2	29	34		
Tumor diameter (cm)				
≥ 2	65	35	8.597	0.003
< 2	98	21		
Pathological type				
Endometrioid adenocarcinoma	153	41	17.577	< 0.001
Non-endometrioid adenocarcinoma	10	15		
Histological grade				
Moderate–high differentiation	147	35	22.751	< 0.001
Low differentiation	16	21		
Cervical interstitial involvement				
Yes	16	13	6.513	0.011
No	147	43		
Lymph node metastasis				
With	11	10	5.933	0.015
Without	152	46		
Paracervical metastasis				
Yes	7	3	0.108	0.742
No	156	53		
Adnexal metastasis				
Yes	8	2	0.171	0.679
No	155	54		
FIGO staging				
I–II	148	48	1.146	0.284
III–IV	15	8		

*BMI* body mass index, *FIGO* International Federation of Gynecology and Obstetrics.

### Comparison of patient laboratory data

The laboratory indicators of patients with LVSI were compared with those without the training group. The results showed significant statistical differences in lymphocyte count (LYM), monocyte count (MONO), albumin (ALB), fibrinogen (FIB), monocyte-to-lymphocyte ratio (MLR), and platelet-to-lymphocyte ratio (PLR) between the two groups (*P* < 0.001, Table 2).

**Table 2:** Comparison of laboratory indicators between patients with and without LVSI in the training group.

Categories	Non-LVSI group (n = 163)	LVSI group (n = 56)	Z/t	*P*
NEUT (× 10^9^/L)	3.36 ± 1.12	3.56 ± 0.78	− 1.241	0.218
LYM (× 10^9^/L)	1.59 ± 0.46	1.89 ± 0.48	− 4.26	< 0.001
MONO (× 10^9^/L)	0.34 ± 0.08	0.29 ± 0.04	5.000	< 0.001
PLT (× 10^9^/L)	271.57 ± 46.77	261.89 ± 40.49	1.382	0.171
ALB (g/L)	37.71 ± 4.46	41.99 ± 5.21	− 5.932	< 0.001
FIB (g/L)	391.98 ± 65.11	307.91 ± 56.44	8.614	< 0.001
PNI	47.72 ± 5.63	49.34 ± 5.27	− 1.888	0.062
NLR	2.01 [1.54, 2.95]	1.76 [1.47, 2.41]	1.733	0.083
MLR	0.20 [0.17, 0.28]	0.15 [0.12, 0.18]	5.505	< 0.001
PLR	166.05 [127.00, 221.73]	135.71 [113.20, 173.82]	3.633	< 0.001

*NEUT* neutrophil count, *LYM*: lymphocyte count, *MONO*: monocyte count, *PLT*: platelet count, *ALB*: albumin, *FIB*: fibrinogen, *PNI*: prognostic nutritional index, *NLR*: neutrophil-to-lymphocyte ratio, *MLR*: monocyte-to-lymphocyte ratio, *PLR*: platelet-to-lymphocyte ratio.

Normally distributed data are expressed by means ± SD, while non-normally distributed data are expressed by median and interquartile range (IQR).

### Multivariable logistic regression analysis

We assigned values to the indices with differences in the univariate analysis (Table 3), where the metric data were dichotomized using Cut-off values as cut-off points^16^, and then found through multivariable logistic regression analysis that myometrial infiltration depth (P < 0.001, OR = 17.876, 95% CI 5.546–57.619), cervical interstitial involvement (*P* = 0.003, OR = 8.028, 95% CI 2.037–31.639), LYM (*P* = 0.001, OR = 0.159, 95% CI 0.055–0.465), MONO (*P* = 0.001, OR = 0.149, 95% CI 0.048–0.460), ALB (*P* = 0.002, OR = 0.175, 95% CI 0.059–0.524), and FIB (*P* < 0.001, OR = 11.853, 95% CI 4.285–32.788) were independent risk factors for LVSI in EC (Table 4).

**Table 3:** Assignment table.

Factors	Assignment
Myometrial infiltration depth (cm)	< 1/2 = 0, ≥ 1/2 = 1
Tumor diameter (cm)	≥ 2 = 1, < 2 = 0
Pathological type	Endometrioid adenocarcinoma = 1, non-endometrioid adenocarcinoma = 0
Histological grade	Moderate–high differentiation = 1, low differentiation = 0
Cervical interstitial involvement	With = 1, without = 0
Lymph node metastasis	With = 1, without = 0
LYM	≥ 1.755 = 1, < 1.755 = 0
MONO	≥ 0.335 = 0, < 0.335 = 1
ALB	≥ 42.405 = 1, < 42.405 = 0
FIB	≥ 362.165 = 1, < 362.165 = 0
MLR	≥ 0.1778941 = 1, < 0.1778941 = 0
PLR	≥ 202.3810926 = 1, < 202.3810926 = 0
LVSI	LVSI group = 1, non-LVSI group = 0

*LYM* lymphocyte count, *MONO* monocyte count, *ALB* albumin, *FIB* fibrinogen, *MLR* monocyte-to-lymphocyte ratio, *PLR* platelet-to-lymphocyte ratio.

**Table 4:** Multivariable logistic regression.

Factors	β	Standard error	χ^2^	P	OR	95% CI	
						Lower bound	Upper bound
Myometrial infiltration depth	2.883	0.597	23.315	< 0.001	17.876	5.546	57.619
Tumor diameter	− 1.511	1.215	1.547	0.214	0.221	0.02	2.387
Pathological type	− 1.045	0.734	2.023	0.155	0.352	0.083	1.484
Histological grade	− 1.352	1.018	1.766	0.184	0.259	0.035	1.901
Cervical interstitial involvement	2.083	0.700	8.860	0.003	8.028	2.037	31.639
Lymph node metastasis	− 0.166	1.581	0.011	0.916	0.847	0.038	18.785
LYM	− 1.838	0.547	11.306	0.001	0.159	0.055	0.465
MONO	− 1.905	0.576	10.946	0.001	0.149	0.048	0.460
ALB	− 1.741	0.558	9.729	0.002	0.175	0.059	0.524
FIB	2.473	0.519	22.683	< 0.001	11.853	4.285	32.788
MLR	0.601	0.702	0.733	0.392	1.824	0.461	7.222
PLR	0.857	0.696	1.515	0.218	2.356	0.602	9.222

*LYM* lymphocyte count, *MONO* monocyte count, *ALB* albumin, *FIB* fibrinogen, *MLR* monocyte-to-lymphocyte ratio, *PLR* platelet-to-lymphocyte ratio.

### LASSO regression analysis

**Figure 1:**
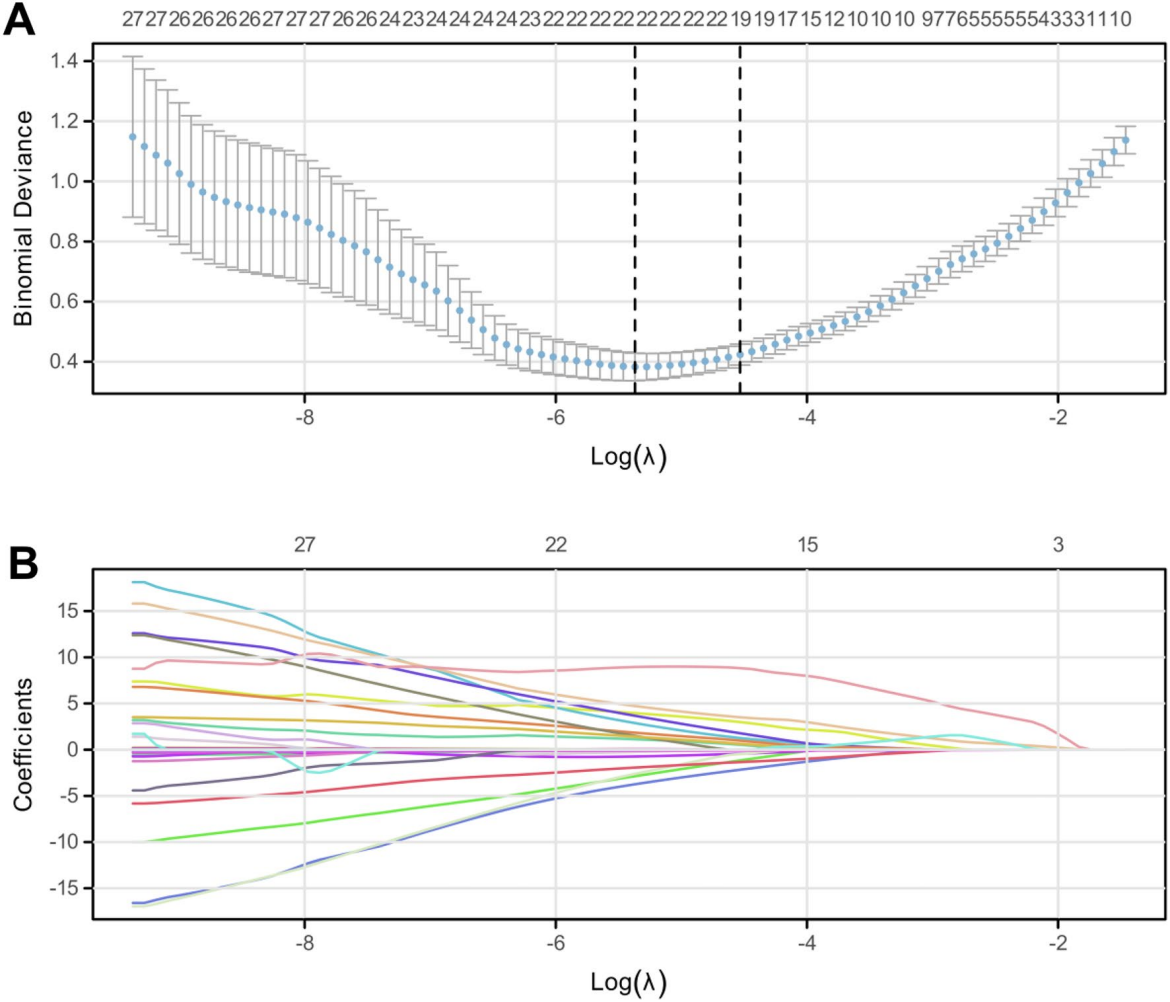
LASSO regression screening for characteristic factors of lymphovascular space invasion in endometrial cancer. (**A**) LASSO regression for the screening of characteristic factors leading to lymphovascular space invasion in endometrial cancer; **(B**) 19 characteristic factors when = λ.1se.

In the present study, we used LASSO regression to screen the characteristic factors leading to LVSI in EC. LASSO regression identified 22 characteristic factors when = λmin (0.0046564) and 19 characteristic factors when = λ0.1se (0.010757) (Fig. 1A). Considering the generalization performance of the model, 19 characteristic factors were identified when = λ0.1SE was selected to construct the model. A total of 19 characteristic factors were screened out, including age, menarche, menopause, gravidity, parity, history of diabetes, myometrial infiltration depth, tumor diameter, pathological type, histological grade, cervical interstitial involvement, adnexal metastasis, FIGO staging, NEUT, LYM, MONO, ALB, FIB, and PLR (Fig. 1B).

### Risk model construction

**Figure 2:**
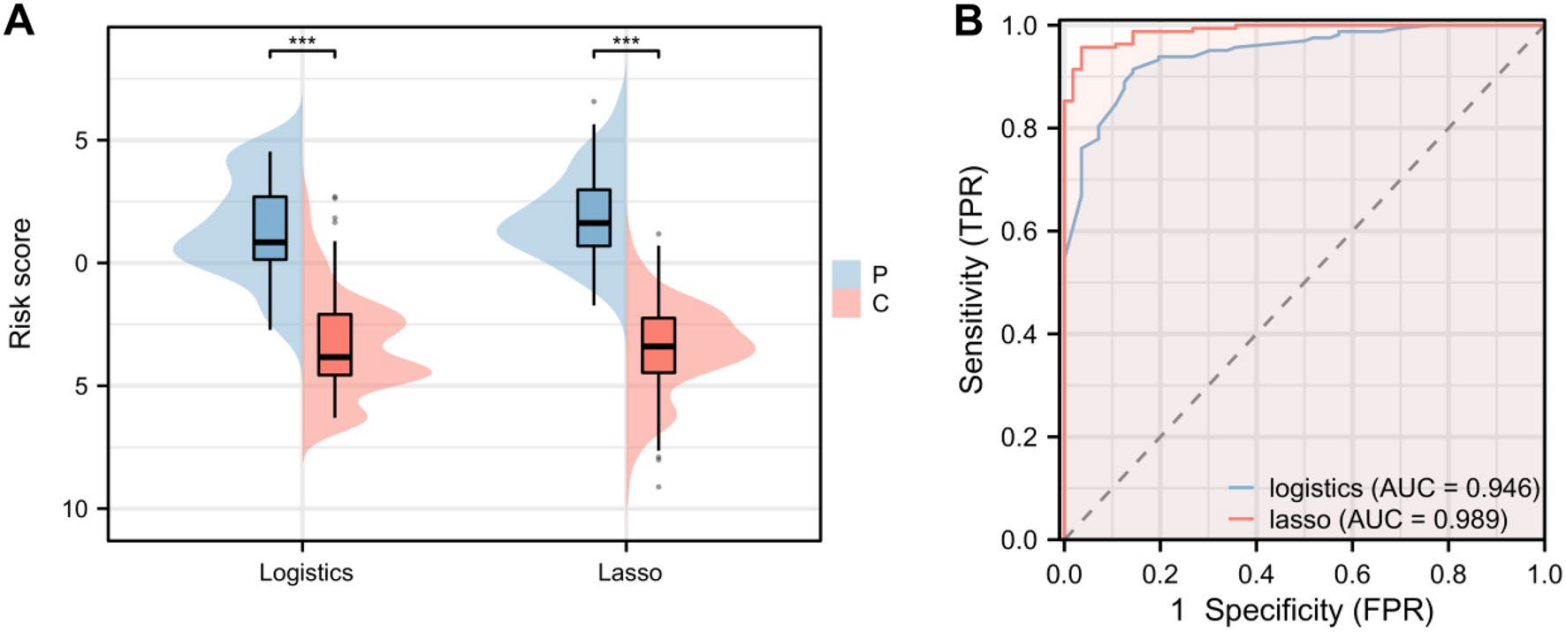
Comparison of risk scores and predictive efficiency between LVSI and non-LVSI patients in the training group. (**A**) Comparison of the patient scores in the training group calculated by logistic regression and LASSO regression. (**B**) ROC curve analysis of the AUCs of logistic regression and LASSO regression risk scores in predicting lymphovascular space invasion in endometrial cancer. *Note* LVSI: lymphovascular space invasion; ROC: receiver operating characteristic; AUCs: areas under the curves; C represents the non-LVSI group and *P* represents the LVSI group.

We constructed two risk models by logistic and LASSO regression. The logistic regression models were constructed using the β coefficient (Table 4), and the LASSO regression also used this coefficient construction model (Table 5). By comparison, we found that in both models, the risk score of patients in the LVSI group was significantly higher compared with that of patients in the non-LVSI group in the training group, with a statistical difference (*P* < 0.001, Fig. 2A). Through Delong test analysis, the area under the curve (AUC) of the risk model, which was built based on logistic regression was significantly lower compared with that of the LASSO-constructed model (*P* < 0.001, Fig. 2B).

**Table 5:** LASSO characteristic variables.

variable	lambda.1se
(Intercept)	− 6.940739155
Pathology type	− 2.169278799
LYM	− 1.342197496
Menopausal status	− 1.158555229
Adnexal metastasis	− 0.470917304
Histologic grading	− 0.431872575
ALB	− 0.078964456
First menstrual period	− 0.030405188
NEUT	− 0.026419354
PLR	0.000390068
Age	0.003689323
FIB	0.01601411
Tumor diameter	0.530228619
FIGO staging	0.632008205
Cervical mesenchymal involvement	0.974989372
Frequency of delivery	1.261055354
History of diabetes	1.778972607
Frequency of pregnancy	2.987453542
depth of myometrial infiltration	3.508977168
MONO	8.82407475

### Modeling validation

**Figure 3:**
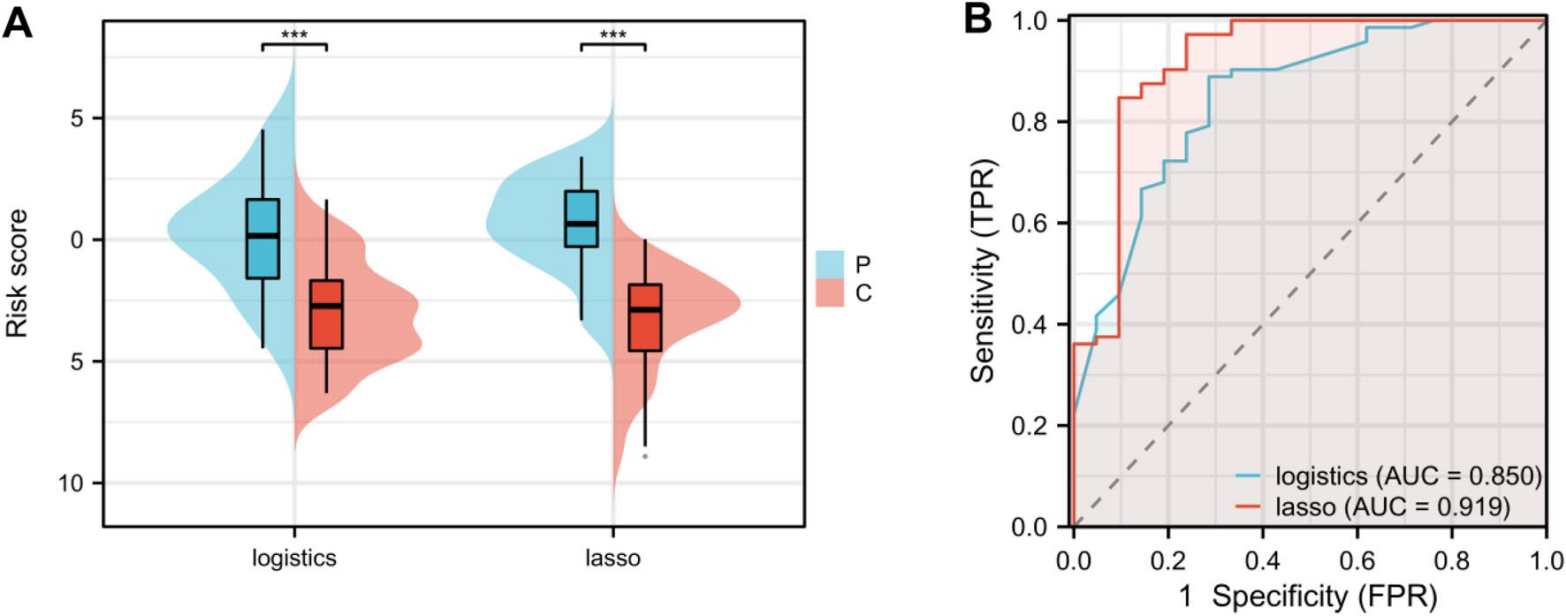
Comparison of risk scores and predictive efficiency between LVSI and non-LVSI patients in the validation group. (**A**) Comparison of the risk score of patients in the validation group calculated by logistic regression and LASSO regression; (**B**) ROC curve analysis of the AUCs of logistic regression and LASSO regression risk scores in predicting lymphovascular space invasion in endometrial cancer. *Note* LVSI: lymphovascular space invasion; ROC: receiver operating characteristic; AUCs: areas under the curves; C represents the non-LVSI group and *P* represents the LVSI group.

A comparison of patient baseline data between the training and validation groups revealed no statistical difference between them (*P* > 0.05, Table 6). We calculated the logistic and LASSO risk scores of patients in the validation group. LVSI patients had higher logistic and LASSO risk scores compared with the non-LVSI patients in the validation group, with significant statistical differences (*P* < 0.001, Fig. 3A). Subsequently, we found through the Delong test that the AUC of the logistic regression risk model was significantly lower compared with that of the LASSO risk model (*P* < 0.001, Fig. 3B, Tables 7, 8).

**Table 6:** Comparison of the clinical data between training and validation groups.

Factors	Training group (n = 219)	Validation group (n = 93)	t/χ2	P
Age (years)	57.41 ± 9.66	57.42 ± 8.96	− 0.006	0.995
BMI (kg/m^2^)	25.77 ± 2.58	25.78 ± 2.53	− 0.019	0.985
Menarche (years of age)	14.00 [13.00, 15.00]	14.00 [13.00, 15.00]	− 0.029	0.977
Menopause				
Yes	70	32	0.177	0.674
No	149	61		
Gravidity				
≥ 2	145	60	0.083	0.773
< 2	74	33		
Parity				
Multipara	169	69	0.319	0.572
Primipara	50	24		
History of hypertension				
With	66	29	0.034	0.854
Without	153	64		
History of diabetes				
With	65	30	0.205	0.651
Without	154	63		
Myometrial infiltration depth (cm)				
< 1/2	63	24	0.285	0.594
≥ 1/2	156	69		
Tumor diameter (cm)				
≥ 2	100	48	0.927	0.336
< 2	119	45		
Pathological type				
Endometrioid adenocarcinoma	194	80	0.401	0.527
Non-endometrioid adenocarcinoma	25	13		
Histological grade				
Moderate–high differentiation	182	77	0.004	0.947
Low differentiation	37	16		
Cervical interstitial involvement				
Yes	29	13	0.030	0.862
No	190	80		
Lymph node metastasis				
Yes	21	10	0.099	0.753
No	198	83		
Paracervical metastasis				
Yes	10	5	0.094	0.760
No	209	88		
Adnexal metastasis				
Yes	10	6	0.477	0.490
No	209	87		
FIGO staging				
I–II	196	82	0.118	0.731
III–IV	23	11	0.177	0.674
NEUT (× 10^9^/L)	3.54 ± 0.78	3.51 ± 0.88	0.368	0.713
LYM (× 10^9^/L)	1.76 ± 0.45	1.81 ± 0.49	− 1.032	0.303
LYM (× 10^9^/L)	0.29 [0.26, 0.32]	0.29 [0.26, 0.33]	− 0.853	0.393
PLT (× 10^9^/L)	266.18 ± 42.95	264.37 ± 42.29	0.343	0.732
ALB (g/L)	41.23 ± 5.57	40.89 ± 5.35	0.489	0.626
FIB (g/L)	315.24 [278.23, 364.60]	322.44 [280.75, 374.39]	− 1.012	0.312
PNI	49.84 ± 5.31	48.92 ± 5.40	1.388	0.167
NLR	2.01 [1.63, 2.47]	1.82 [1.48, 2.55]	1.347	0.178
MLR	0.17 [0.14, 0.20]	0.16 [0.13, 0.21]	0.610	0.542
PLR	153.85 [128.73, 177.14]	140.71 [118.21, 181.88]	1.338	0.181

*BMI* body mass index, *FIGO* International Federation of Gynecology and Obstetrics, *NEUT* neutrophil count, *LYM* lymphocyte count, *MONO* monocyte count, *PLT* platelet count, *ALB* albumin, *FIB* fibrinogen, *PNI* prognostic nutritional index, *NLR* neutrophil-to-lymphocyte ratio, *ML*R monocyte-to-lymphocyte ratio, *PLR* platelet-to-lymphocyte ratio.

Normally distributed data are expressed by means ± SD and non-normally distributed data by IQR.

**Table 7:** ROC Curve parameters.

Marker	AUC	95% CI	Specificity (%)	Sensitivity (%)	Youden index (%)	Cut off (%)	Accuracy (%)	Precision (%)	F1 Score (%)
Training group-logistics	0.946	0.916–0.946	91.41	85.71	77.13	− 0.366	89.95	85.71	81.36
Training group-lasso	0.989	0.980–0.989	95.71	96.43	92.13	− 0.633	95.89	96.43	92.31
Validation group-logistics	0.85	0.754–0.850	88.89	71.43	60.32	− 0.048	84.95	71.43	68.18
Validation group-LASSO	0.919	0.836–0.919	84.72	90.48	75.20	− 1.38	86.02	90.48	74.51

**Table 8:** Delong test parameters.

Marker1	Marker2	Z value	P value	AUC difference	95%CI
Training group-logistics	Training group-LASSO	− 3.36	< 0.001	− 0.044	− 0.069 to − 0.018
Validation group-logistics	Validation group-LASSO	− 2.157	0.031	− 0.070	− 0.133 to − 0.006

## Discussion

Many scholars believe that LVSI is the basis for determining whether tumor cells have metastasized to lymph nodes^17^. LVSI is defined as at least one cluster of tumor cells that is observed to gather in the gap of the enveloping layer of flat endothelial cells and attach to the blood vessel wall by observing conventional pathological sections after surgery with an optical microscope^18^. The formation of LVSI is a complex pathophysiological process. When tumor cells penetrate the basement membrane and invade or penetrate into the surrounding tissues, it is usually accompanied by the invasion of interstitial lymphatic vessels and small vessels^11^. Tumor cells invading these lymphatic vessels or small blood vessels can form homopolymers, or bind to white blood cells and platelets to form heteropolymers, which results in intravascular cancer thrombosis^19^. Furthermore, these tumor thrombi float in blood vessels or lymphatic vessels and spread through the bloodstream or lymphatic system to all parts of the body to promote tumor metastasis.

Clinical prediction models serve as essential quantitative tools for assessing risks and benefits, offering convenient, intuitive, and accurate insights for healthcare professionals and policymakers^20^. These models use multivariable regression analysis to integrate multiple predictors, enabling the quantitation of risks and the evaluation of prognosis for a variety of cancers. For example, Zuber et al.^21^ developed a model predicting OS in adrenocortical carcinoma patients, achieving AUCs between 0.68 and 0.72 for the training and validation sets for 5-years and 10-years OS. Similarly, another study^22^ constructed a nomogram to predict 3-years survival post-radical resection in colon cancer patients, and achieving a high C-index of 0.918. In addition, an EC prognostic model^23^ was formulated based on inflammatory response-related genes (IRGs), and 13 IRGs were identified as independent prognostic markers capable of predicting survival and response to chemotherapy and immunotherapy. In the present study, logistic regression analysis pinpointed independent risk factors for LVSI in EC, including myometrial infiltration depth, cervical stromal invasion, LYM, MONO, ALB, and FIB. These factors underlie the link of LVSI to various biological processes and immune status. Increased myometrial infiltration depth suggests tumor spread to deeper tissues, heightening the risk of lymphatic vasculature invasion^24^. Cervical stromal invasion indicates local tumor spread, potentially increasing the risk of lymphatic system invasion. Alterations in LYM and MONO levels indicate immune response variations, possibly because of changes in the tumor microenvironment that facilitate tumor cell lymphatic dissemination^25^. Furthermore, changes in ALB and FIB levels may signal inflammation and coagulation mechanisms involved in tumor aggressiveness and metastasis^26,27^. Risk scores, calculated using β coefficients as risk indicators, identified LVSI patients in both training and validation groups with significantly higher scores compared with their non-LVSI counterparts. ROC curve analysis further affirmed the high predictive value of the logistic risk model, with AUCs of 0.946 and 0.850, respectively, which demonstrate the exceptional accuracy of the model at predicting LVSI in EC.

Although logistic regression models are efficient and robust in predicting LVSI in EC, they may not fully capture the complex, nonlinear relationships inherent to EC-LVSI. Recent studies, including Shao et al.^28^, have demonstrated the superiority of LASSO over logistic regression in disease prediction. For example, LASSO was superior for predicting diabetic foot ulcer progression in elderly diabetic patients. Similarly, we found that LASSO outperformed logistic regression in predicting LVSI in EC. LASSO identified 19 key factors related to LVSI, including patient age, menstrual history, tumor pathology, and hematological markers. This underscores the multifactorial nature of LVSI and the ability of LASSO to comprehensively capture these complex interactions via feature selection. The LASSO model consistently exhibited higher accuracy for both the training and validation groups compared with logistic regression. This aligns with the findings that LASSO can generate more accurate and robust prediction models by selecting robust features and eliminating non-contributory ones through coefficient regularization, thereby mitigating model overfitting.

This study has some limitations. Because it was single-center and retrospective, this study had some typical limitations, such as a small sample size and selection bias, which limits further improvement of the prediction efficiency of the model and affects the interpretation of the results. In the future, we will validate and optimize the model using a prospective multi-center, large-sample random sampling research design, that focuses on monitoring the stability of the model in independently validated samples. The results provide a foundation for feature selection and model establishment for the prediction of LVSI in EC.

## Conclusion

Overall, this study demonstrated the feasibility of the LASSO method to establish a more accurate and reliable prediction model for LVSI in EC. It combines the clinical features, tumor characteristics, and hematological indicators of patients to reflect the high-risk factors of LVSI from multiple aspects. The application of this prediction model can help doctors identify patients with high LVSI risk earlier and adjust their treatment plan accordingly to obtain a better prognosis.

## Data Availability

The data used in the above analysis are available upon reasonable request from the corresponding author.
